# Critical temperature, surface tension and vapor density estimations of methanol using the scaled model

**DOI:** 10.1016/j.heliyon.2019.e01595

**Published:** 2019-05-13

**Authors:** Abdalla Obeidat, Mohammad Badarneh

**Affiliations:** Jordan University of Science and Technology, Physics Department, Irbid 22110, Jordan

**Keywords:** Condensed matter physics, Molecular physics, Statistical physics

## Abstract

In the present work, we applied scaled model to calculate surface tension, vapor densities and the critical temperatures of four different models of methanol: namely, H1, J1, J2 and L1 models. The scaled model is based on calculating the free energy of the system. Free energy calculations were performed by applying the Bennet acceptance ratio (BAR) using Monte-Carlo simulations at low temperature range of 220K–280K. The BAR is based on calculating the free energy difference of n-molecules and (n-1)-molecules plus a free probe on methanol. Estimations of vapor densities are based on extrapolating the intercept of the scaled free energy linear line as number of molecules approaches infinity, which requires a pre-known values for liquid densities. To accomplish this, a series of molecular dynamic simulations were performed at low temperature range of 200K–300K with steps of 10K. All the estimated properties were in excellent agreement with experimental published data.

## 1 Introduction

Due to importance of methanol in our daily life and its applications in engineering, science, medicine and industry [1, 2, 3, 4], scientists investigated and will continue investigate this substance experimentally and theoretically at macro and micro levels [5, 6, 7, 8, 9, 10, 11, 12, 13]. Methanol is one of simplest polar fluids which consists of methyl group (CH3) and hydroxyl group (OH) and due to is availability, computational scientists studied this molecule extensively. The most important criterion to computational scientists is finding a potential model for the molecule and a method to calculate and estimate the properties of the substance. The potential model depends on the partial charges on the atoms and on the Lennard-Jones parameters; the segment diameter and the strength of the well. Many scientists propose different potential functions for methanol, some of these potentials are based on six-site atoms such as OPLS-AA [14] and GROMOS96 [15], and some are based on three-site atoms by assuming the methyl group as a unite atom and in this case the methanol computationally looks like water with different parameters such as H1, H2 [16], J1, J2 [17], B3 [18], L1 [19] and TraPPE-UA [20]. On the other hand, the model depends on the method of simulation and these simulations could be standard to computational community, or might be a rigid model based on applying a statistical mechanics theory in nonstandard method.

The standard method of computing vapor and liquid densities and estimating surface tension as well using molecular dynamics and Monte-Carlo simulations is by placing the substance in a slab between to vacuum boxes to ensure surfaces. Vapor and liquid densities are then estimated by fitting the density profiles to tangent hyperbolic or error functions [21, 22]. The problem with this method is its failure of estimating vapor density at low temperatures since at these temperatures the number of molecules in the vacuum is almost null. For this reason, the fitting parameter for vapor density is set to zero. Regarding surface tension calculations, the method is also standard but with different approaches of calculations. One method of calculations is based on evaluating the components of pressure tensor as in [23, 24], better estimation is by taking into account the tail correction for the Lennard-Jones interaction [25, 26]. Another standard method is applying the theories of Kirkwood and Buff by calculating the total forces [27]. Recently, Vega and de Miguel [28] calculated the surface tension by applying the test-area method proposed by Gloor et al [29]. The method is based on the definition of surface tension by calculating the change in free energy associated with small change in the interfacial area at constant volume.

The critical temperature *T*_c_, however, is not easy to estimate, one of the best methods to estimate it is by calculating the vapor-liquid phase diagram as a function of temperature *T* and making a fit to the points using Wegner expansion with constants depends on the substance [30]. Another elegant method is by imposing Maxwell construction to an equation of state and make a fit to the phase diagram [31], or using the compressibility factor after estimating an equation to the coexistence curve to generate the coefficients required to approximate the critical temperature [32]. An easy method [33, 34] might be through fitting the surface tension to $\frac{T_{c}}{T}-1$ with exponent of unknown value of $T_{c}$, while the easiest method is by calculating surface tension as a function of temperature at relatively high temperatures then extrapolate the critical temperature by making a linear fit, the point at which the linear curve intersects with the temperature coordinate is the critical temperature since at this point the surface tension is zero [35].

In our previous work, we estimated the critical temperature using four different models of water and the critical temperatures we found were overestimated in all models [36]. On the other hand, the estimation of surface tension and vapor density were in good agreement with experimental data at low temperatures. Also, surface tension estimation values were in good agreement with experimental data at high temperatures as well even though all the simulations were performed at low temperatures. In this paper, we used scaled model by Hale [37] to estimate vapor density, surface tension and the critical temperature for the four different methanol models, specifically, H1, J1, J2 and L1. The scaled model is based on calculating the free energy difference between two systems; the first system consists of n−1 interacting molecules and a free probe while the second system consists of n interacting molecules. These systems are composed of clusters with small number of molecules placed inside a sphere or a box, in contrast to the standard method of calculating the above thermodynamic properties where one needs to make an interface with a relatively high number of molecules to form a bulk. The free energy difference at different temperatures is then scaled to $\frac{T_{c}}{T}-1$ where $T_{c}$ can be treated as a variable that makes all the calculated free energy difference collapse into a single line. Changing the $T_{c}$ variable will give an approximate value for the critical temperature. The slope of the line for scaled free energy difference of clusters consist of eight molecules and higher is related to the surface tension at which one can estimate the surface tension. Finally, the extrapolate value of the intercept of the line is related to the ratio of liquid density to that of vapor density. The value of vapor density is then calculated with pre-known value of liquid density; these pre-known values have been estimated by applying the standard method mentioned above using molecular dynamic simulations. On the other hand, the free energy difference is calculated based on the Bennett acceptance ratio [38] using Monte-Carlo simulations. It is worth mentioning that the scaled model is a phenomenological model based in assuming capillarity approximation for the cluster free energy for large enough cluster sizes, with a surface tension that is linear in temperature and vanishing at the critical temperature. So, this method is not applicable for all substance, but with those follow capillarity approximation such as water and alcohols.

## 2 Theory

As pointed out by Shirts et al [39, 40] that the most efficient method of calculating the free energy difference of states is the Bennett acceptance ratio (BAR), since all other methods like exponential averaging (EXP) [41], thermodynamic integration (TI) [42], umbrella sampling (US) [43] and umbrella integration (UI) [44], and weighted histogram analysis method (WHAM) [45] lack a standard test. We followed the same scheme as in our previous work in applying scaled model, which requires free energy calculations, with the aid of BAR. The BAR attracted many scientists and was applied in different areas of research [46, 47, 48]. In this method, the free energy difference between the two states is given by:

(1) $$\Delta F=-k_{B}Tln\frac{\langle f\left(-\beta \left(\Delta V-C\right)\right)\rangle }{\langle f\left(\beta \left(\Delta V-C\right)\right)\rangle }\;+C$$

where $\beta =1/k_{B}T$, *k*_*B*_ is the Boltzmann constant and C is an arbitrary number, and $f\left(x\right)=\frac{1}{1+e^{x}}$ is the Fermi function. The optimum value for C has proven to be when the ratio of the averaging Fermi functions are equal, leaving us with ΔF=C.

In our simulation, we used the same number of independent configurations, i.e., $N_{A}=N_{B}$, and in this case the variance of the free energy difference is given by

(2) $$\sigma^{2}=\frac{{\langle f^{2}\rangle }_{B}-{\langle f\rangle }_{B}^{2}}{{\langle f\rangle }_{B}^{2}}+\frac{{\langle f^{2}\rangle }_{A}-{\langle f\rangle }_{A}^{2}}{{\langle f\rangle }_{A}^{2}}$$

The Scaled model is based on law of mass action, we are following the scheme of Hale and Ward [49], which depends on calculating the free energy difference between two systems: the first one consists of n−1 molecular cluster with a free monomer that interacts very weekly with the cluster called the probe, and we call this system ensemble A. The second system consists of n molecular cluster, named ensemble B. The total interaction of ensemble A is $V_{A}=\frac{V+\lambda \Delta V}{k_{B}T}$, while for ensemble B is $V_{B}=\frac{V+\Delta V}{k_{B}T}$, where V is the interaction energy of all the n−1 moleculare cluster, ΔV is the interaction of the probe with n−1 molecule cluster, and λ is a very small number; in our simulation, we used $\lambda =10^{-8}$. This value has been tested with other values, the choice of λ depends on the efficiency of the Bennet technique which relies on an adequate overlap between the energy of the two ensembles, and this achieved by choosing small values of λ. Hale in her analysis showed that the difficulty of connecting the optimal value of C to the free energy difference comes from the simulation volume of the free probe $V_{n}$. In her analysis, she assumed that the volume is given by $V_{n}=\alpha \left[\frac{n}{\rho_{l}}\right]$, where α should be kept constant on all the simulations as proposed by Lee et al [50], and n is the number of molecules in the cluster. In other words, α is the ratio of the simulated volume to the volume of the n molecules. In this work, we performed a set of computational experiments by varying α to be 5, 6, and 7 for small clusters of 2, 3, 4, 5, 6, 7, and 8 L1-methanol molecules at T=260K and T=280K, all the calculated free energies at specific number of molecules were similar regardless of the value of α, we fixed α to be 5. Following these analysis, Hale argued that the difference in free energy between ensembles A and B is given by:

(3) $$-\delta F_{n}=\ln\left[\frac{Q\left(n\right)}{Q\left(n-1\right)Q\left(1\right)\left(\frac{\alpha V_{n}}{V}\right)}\right]+ln\alpha =C_{optimal}\left(n\right)+ln\alpha$$

where $\delta F_{n}$ is the free energy difference divided by $k_{B}T$. As explained by Hale and DiMattio [51], the free energy difference can be written as

(4) $$-\delta F_{n}=I_{0}-\frac{2}{3}An^{-\frac{1}{3}}$$

where $I_{0}$ equals to $\text{ln}\left(\frac{\rho_{l}}{\rho_{v}}\right)$, here $\rho_{v}$ is vapor density. A is related to the surface tension (γ) and to the entropy per molecule (Ω) as $A={\left(36\pi \right)}^{\frac{1}{3}}\text{Ω}\left[\frac{T_{c}}{T}-1\right]=\frac{{\left(36\pi \right)}^{\frac{1}{3}}\gamma }{k_{B}T\rho_{l}^{\frac{2}{3}}}$, where $T_{c}$ is the critical temperature. So, if −δF is plotted versus $n^{-\frac{1}{3}}$, then $I_{0}$ would represent the extrapolation at which the value of $\rho_{v}$ could be estimated, and the slope would be $-\frac{2}{3}A$ at which surface tension and entropy per particle can be estimated. For more details about the BAR and the law of mass action, please refer to ref [51].

## 3 Methodology

We followed the same method of simulations as in our unpublished work, and here is a summary of the details. The Monte Carlo (MC) simulation has been applied to calculate the free energy differences using the BAR method. We wrote a code for four different models of methanol. The simulation volume is 5 times the volume of the molecules as pointed earlier. The molecules are inserted in a sphere with no cutoffs, i.e., every molecule interacts with the rest of molecules. As a rule of thumb, we equilibrate the system with $\frac{n+1}{3}10^{7}$ steps, and the MC steps are $2n10^{7}$ steps. The step move for small number of molecules is 1Å and the rotation angle is 0.5° about the center of mass of the molecule, while for large number of molecules (>20) the step move is 0.5Å and the rotation angle is 0.25°. In our simulation, we move and rotate all the molecules in each step at the same time and keep the center of mass of the volume fixed. For the B ensemble, we treat each molecule as a probe; in this case averaging over the Fermi function will produce better results instead of assigning only one molecule to be a probe. In all MC simulations, the number of acceptance steps of 40%−60% is achieved even though this condition is not a necessity as pointed by Landau [52]. Also we applied molecular dynamics simulation using GROMACS package [53] to calculate the liquid density of the four models of methanol at all temperatures between 200K and 300K with step of 10K. This value is very important to calculate vapor density, since the intercept is proportional to the logarithmic function of the ratio of liquid density to that of the vapor density when plotting the free energy difference versus the number of molecules to the power minus one-third. Each system is equilibrated for 1ns using NPT ensemble. For NPT we used Berendsen algorithm [54] where the temperature and the pressure is kept at 1 atm, the time step used is 1fs. After applying NPT for 1ns, the system is then equilibrated for another 1ns using NVT ensemble with Nose-Hoover thermostat [55, 56]. The data then are collected for another run of 4ns. In our simulations, periodic boundary conditions are used in all dimensions, and the size of our system is 1000 molecules.

## 4 Results and discussion

**Fig. 1:**
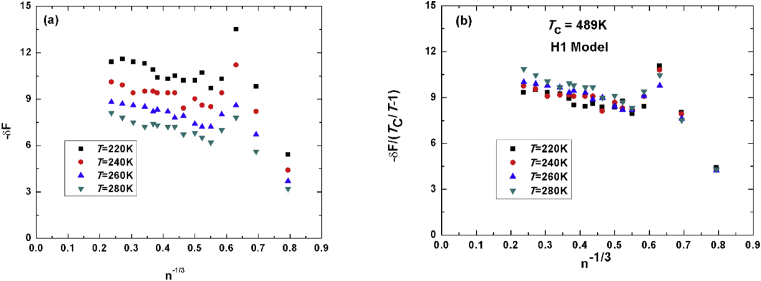
(a) unit less energy versus minus third root of number of molecules. (b) scaled free energy to $\frac{T_{c}}{T}-1$ with theoretical value of H1-methanol of 489K.

Fig. 1a shows the unit less free energy of H1-methanol potential function as a function of number of molecules to the power of minus one-third in a cluster consists of 2, 3, 4, 5, 6, 7, 8, 9, 10, 11, 12, 13, 14, 15, 18, 20, 25, 50, and 75 molecules at four different temperatures: 220K, 240K, 260K and 280K. All the simulations were performed with same number of molecules and same set of temperatures except for L1. The figure shows a behavior of straight line for n≥8 as the theory predicts. Fig. 1b shows the free energies scaled to the critical temperature $T_{c}$ as $\left(T_{c}/T-1\right)$ versus n^−1/3^ for the data in Fig. 1a, where $T_{c}$ is taken equals to the theoretical value of the H1-methanol (489K). In this experiment we varied $T_{c}$ till all the lines collapse into a single line within the error, and our estimated value for the critical temperature is 492K which is very close to the true value of the model. The estimation error has been calculated using Eq. (2) and all the calculated error for all tested models lies within 1%–3%.

**Fig. 2:**
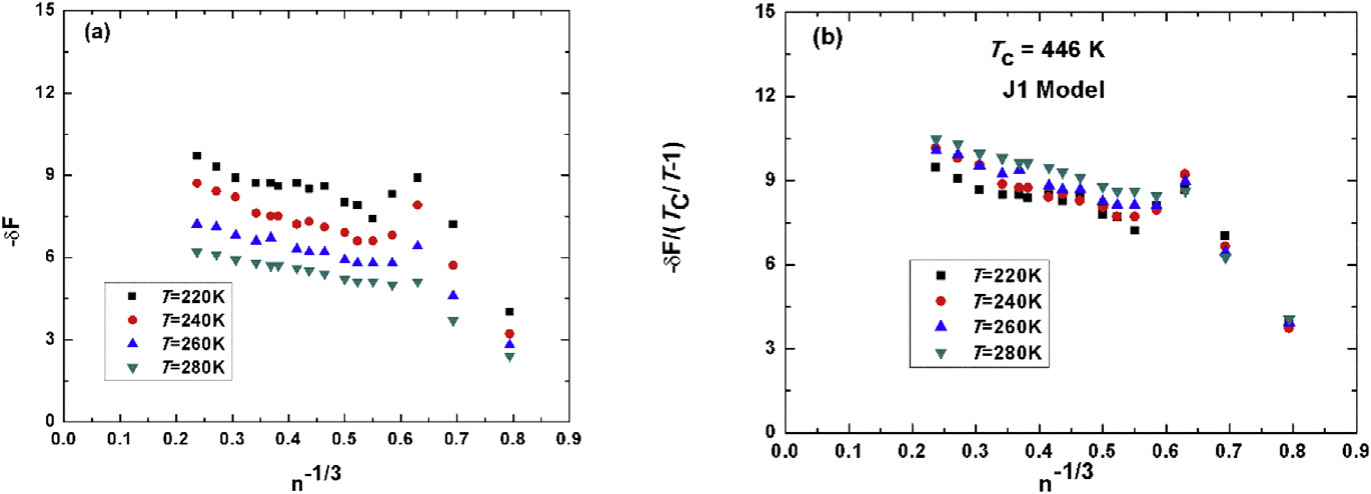
(a) unit less energy versus minus third root of number of molecules. (b) Scaled free energy to $\frac{T_{c}}{T}-1$ with theoretical value of J1-methanol of 446K.

Fig. 2a shows the same as Fig. 1 for J1-methanol potential function with the same number of molecules in the cluster and with the same four different temperatures. The estimated critical temperature is found to be 449K while the theoretical value is 446K.

**Fig. 3:**
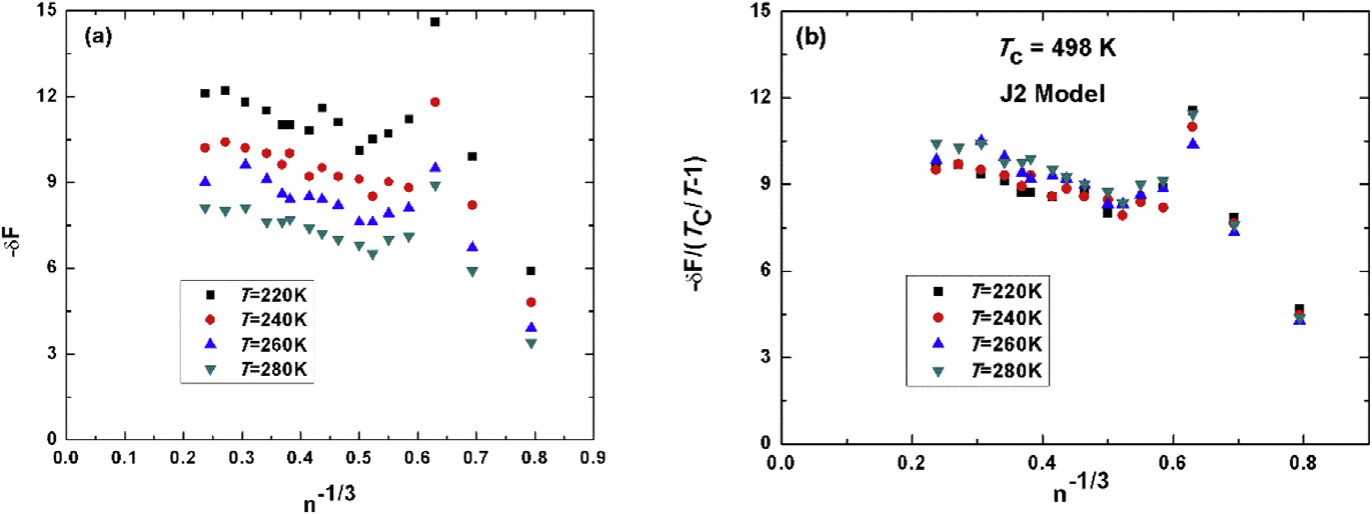
(a) unit less energy versus minus third root of number of molecules. (b) scaled free energy to $\frac{T_{c}}{T}-1$ with theoretical value of J2-methanol of 498K.

For J2-methanol potential function model we present our results in Fig. 3. Fig. 3a shows the free energy as a function of number of molecules raised to the power minus one-third as in Figs. 1a and 2a, while Fig. 3b shows the scaled unitless free energy to $\frac{T_{c}}{T}-1$. The estimated value for the critical temperature in this model is 501K which overestimate the true value of the model of 490K.

**Fig. 4:**
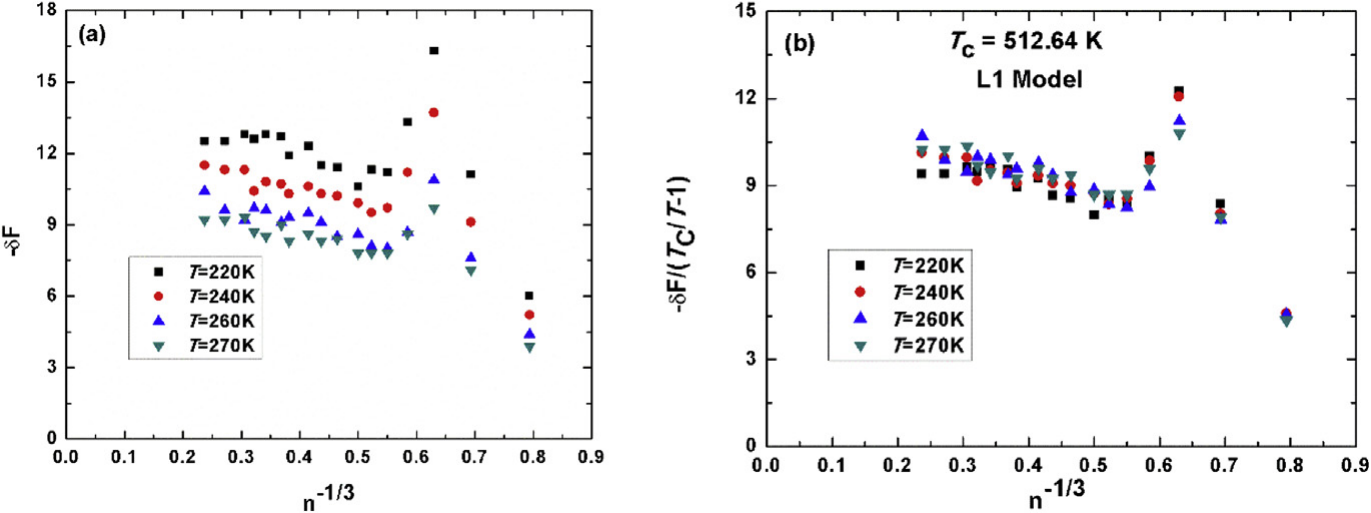
(a) unit less energy versus minus third root of number of molecules. (b) scaled free energy to $\frac{T_{c}}{T}-1$ with theoretical value of L1-methanol of 512K.

Our last example is dedicated to van Leewen and Smit (L1) model, which is the only model that predicts the experimental value of methanol of 512K. Fig. 4a shows the free energy as a function of number of molecules raised to the power minus one-third at *T* = 220K, 240K, 260K and 270K. we notice that the free energy at *T* = 260K and *T* = 270K are very close to each other, that is why we performed our simulations in the other examples at a set of four different temperatures with step of 20K. The scaled free energy to $\frac{T_{c}}{T}-1$ is plotted in Fig. 4b. Our estimated value for the critical temperature of this model is 515K which is very close to the experimental value.

**Fig. 5:**
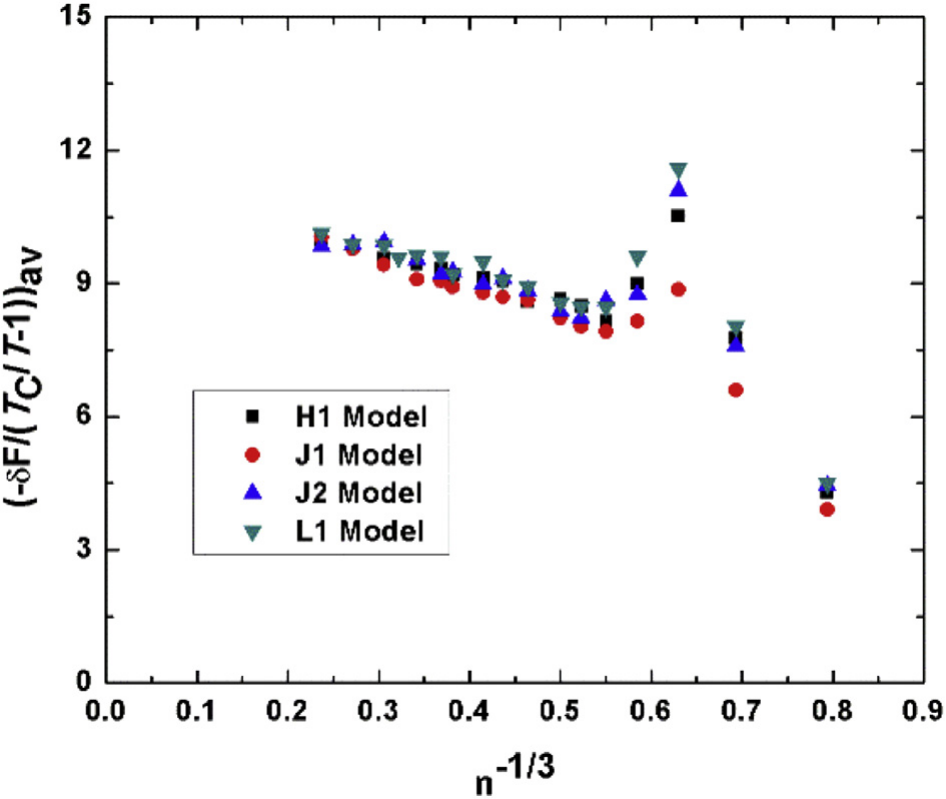
Scaled free energy to $\left(\frac{T_{c}}{T}-1\right)$ of all models.

Fig. 5 shows the average values of the scaled free energy (average values of Figs. 1b, 2b, 3b and 4b) of all models when plotted versus $n^{-\frac{1}{3}}$. As we see from the figure all the scaled free energy of all models coincide on the same line for n > 8 as the theory predicts.

**Fig. 6:**
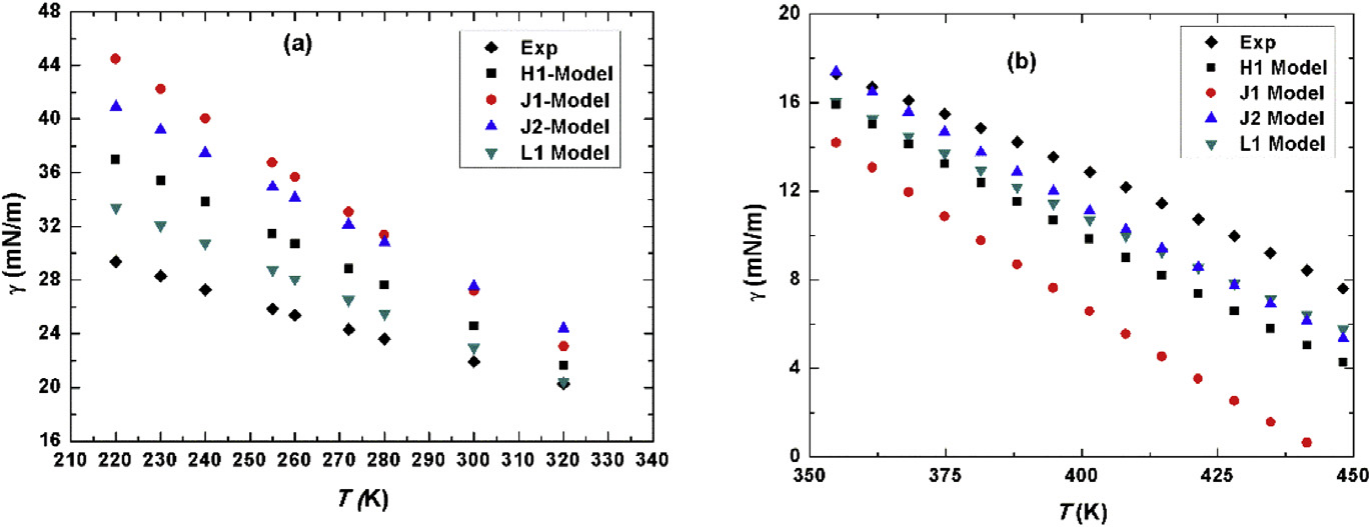
(a) surface tension in units of mN/m of all models versus *T* on the range of 200–300K. (b) same as (a) but at high temperatures.

Fig. 6 shows the calculated values of surface tension in the range of 200K–300K with step of 10K as in Fig. 6a, while Fig. 6b shows our estimated values of surface tension at high temperatures compared to experimental data. At low temperatures, the best model that represent the experimental data is L1 model followed by H1 then J2 whereas J1 shows the worst prediction. On the other hand, at high temperatures we notice that L1 and J2 coincide for *T* > 400K and are very close to experimental results. It is worth mentioning that our results are very good at high temperatures even though our simulations were performed at *T* < 300K. In fact, this is the strength of scaled model where one does not need to perform the simulations at required temperatures, rather one needs only to perform the simulations at relatively three different low temperatures to assure that the molecules stay most of the time close to each other and after scaling the free energy one is able to predict the thermodynamic property at any other temperature.

**Fig. 7:**
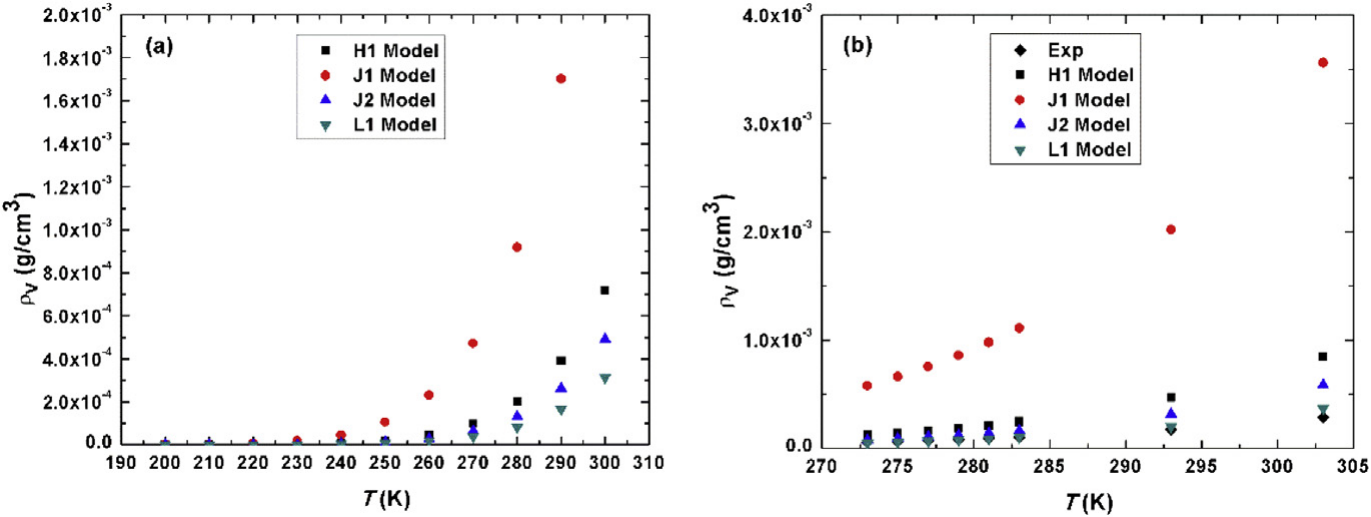
(a) Vapor density in units of g/c,^3^ of all models versus *T* on the range of 200–300K. (b) same as (a) but at different temperatures compared to experimental data.

As mentioned in the introduction, scaled model might be the only model that is capable of estimating vapor density using molecular dynamic or Monte-Carlo simulations at low temperatures. All other standard models or methods assume that vapor density is zero at low temperatures, while applying scaled model the intercept of the scaled free energy is proportional to logarithmic ratio of liquid density to vapor density (*I*_*o*_ *= ln(ρ*_*l*_*/ρ*_*v*_). Our estimated vapor densities extrapolated from Figs. 1, 2, 3, and 4 as n→∞ are plotted in Fig. 7. Fig. 7a shows vapor density as a function of temperature for 200K≤T≤300K, while Fig. 7b shows vapor densities of all studied model versus temperature for 0°C≤T≤30°C compared to experimental data, L1 model shows perfect match with experimental results. It should be mentioned that liquid densities have been calculated from density profiles of 1000 molecule placed in a slab inside a box with vacuum on both sides of the slab using GROMACS package. The molecular dynamics simulations we performed at all temperatures between 200K and 300K with step of 10K which are needed to estimate the values of vapor density as in Fig. 7a. On the other hand, we used experimental values of liquid densities in estimating vapor densities as in Fig. 7b.

**Fig. 8:**
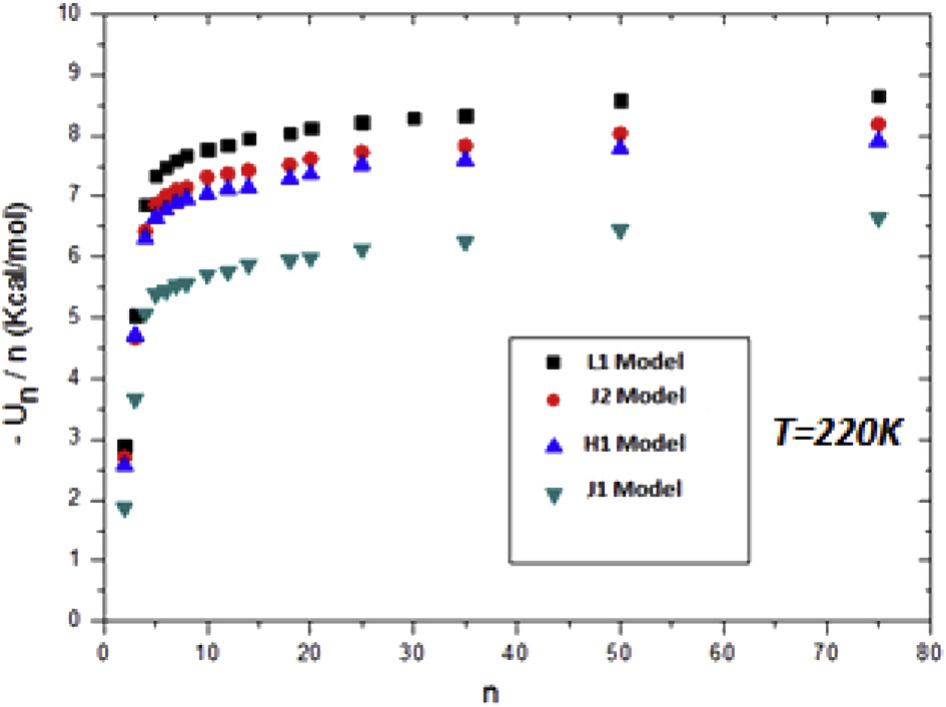
Binding energy of all methanol models at *T* = 220K.

Even though all of the above results show the superiority of L1 model over the others, but we enclose this paper by one last figure about the binding energy of the models at *T* = 220K. As we see from Fig. 8, one might judge the best model among the studied models by looking to the minimum binding energy. It is clear that L1 methanol model has the lowest binding energy followed by J1 then H1, and the highest binding energy of all models is the J1 methanol model. Our results show that H1 and J2 models have almost the same binding energies, and this not unexpected since they almost have the same critical temperature and almost the same estimated vapor densities.

## 5 Conclusions

Critical temperature $T_{c}$, surface tension γ and vapor density $\rho_{v}$ have been estimated for H1, J1, J2 and L1 methanol potential models. Scaling model has been applied to estimate the above-mentioned parameters. Even though the scaling depends on $T_{c}$ as a varying parameter and despite its validity at low temperatures, it gives good results at high temperatures as well. Moreover, this scaling behavior reduces the number of simulations and shows the universality of the behavior of these parameters, beside its capability of predicting the above-mentioned properties at any temperature. Moreover, using the scaled model is capable to obtain the vapor density from the extrapolation of scaled free energy versus n^−1/3^ without assuming it zero as in the standard model. The best obtained data was for L1 model as shown from estimating the properties studied in this work. This is not unexpected since the L1 model has the closest critical temperature to the experimental value. From the results, we note that the closest the critical temperature's model to experimental value, the better the estimated properties values. Finally, the scaled model is capable of estimating the thermodynamic properties over a wide range of temperatures.

## Declarations

### Author contribution statement

Abdalla Obeidat: Conceived and designed the experiments; Wrote the paper.

Mohammad Badarneh: Performed the experiments; Analyzed and interpreted the data.

### Funding statement

This research did not receive any specific grant from funding agencies in the public, commercial, or not-for-profit sectors.

### Competing interest statement

The authors declare no conflict of interest.

### Additional information

No additional information is available for this paper.
